# Post-Market Clinical Follow-Up of the MAX Variable Pitch Compression Screw System in Foot and Ankle Surgery: Safety, Performance, and Patient-Reported Outcomes

**DOI:** 10.3390/jcm15052024

**Published:** 2026-03-06

**Authors:** Thomas J. J. Wolfinger, Séverin R. Wendelspiess, Dirk F. Thümmler, Urs N. Genewein

**Affiliations:** Department of Traumatology, Orthopaedics and Hand Surgery, Fricktal Health Center AG, 4310 Rheinfelden, Switzerland; severin.wendelspiess@unibas.ch (S.R.W.); dirk.thuemmler@gzf.ch (D.F.T.); urs.genewein@gzf.ch (U.N.G.)

**Keywords:** ankle, arthrodesis, bone healing, bone screws, foot, MAX VPC, osteotomy, postoperative complications

## Abstract

**Background/Objectives**: Rigid interfragmentary compression is essential for primary bone healing following fractures, osteotomies, and arthrodeses of the foot and ankle. Evidence on the clinical performance of the MAX Variable Pitch Compression (VPC) Screw System (Zimmer Biomet, Warsaw, IN, USA) remains limited. This post-market, retrospective cohort study evaluated its safety, performance, and patient-reported outcomes. **Methods**: A single-center, consecutive series of patients treated with the MAX VPC Screw System for foot or ankle fractures, osteotomies, or arthrodeses between March 2018 and October 2023 was analyzed. The primary endpoint was radiographic and clinical bone union or joint fusion at 6–8 weeks and ≥18 months. Secondary endpoints included adverse events and functional outcomes using the Foot and Ankle Ability Measure (FAAM). **Results**: A total of 214 procedures were included (27 fractures, 80 osteotomies, 107 arthrodeses). Union was assessed in 209 procedures (97.7%) at 6–8 weeks and in 82 procedures (38.3%) at ≥18 months. Union rates were 86.1% at 6–8 weeks and 98.8% at ≥18 months. Early union was higher in arthrodeses (91.5%) than in fractures/osteotomies (80.6%). Adverse events occurred in 13.1% of procedures, 67.9% of which were device-related; no recurrent mechanical failures were observed. Mean FAAM scores were 92.3 (ADL) and 78.8 (Sports) for arthrodeses and 94.3 and 85.8, respectively, for fractures/osteotomies, at a mean FAAM follow-up of 2.9 years. **Conclusions**: The MAX VPC Screw System demonstrated high bone-union rates, favorable functional outcomes, and a moderate number of device-related complications. These results support its clinical use in foot and ankle surgery. However, the retrospective, single-center design limits generalizability, and prospective multicenter trials are warranted to confirm these findings.

## 1. Introduction

Primary bone healing requires absolute stability with interfragmentary compression [1,2], as applied in fracture stabilization, corrective osteotomies, and arthrodesis. Inadequate stability increases the risk of delayed union, nonunion, hardware failure, and infection [3,4,5,6,7,8], thereby compromising function and outcomes. Fully threaded, variable-pitch headless screws are widely used for fixation in small bones and have been investigated in several studies, particularly with respect to scaphoid fractures [9,10,11,12,13,14,15,16,17,18,19]. Their design, featuring a coarser distal and a finer proximal thread, creates differential advancement of the screw segments during insertion, thereby generating interfragmentary compression [10,12,13,14,15,16,17,18,19] and supporting primary bone healing. Beyond screw design, tightening properties and insertion torque influence the biomechanical stability of screw–bone constructs. Screw tightening in clinical practice is often performed empirically and may result in under- or over-tightening. Moldovan et al. demonstrated that cortical screw insertion follows a reproducible torque profile with a definable optimal tightening window before mechanical failure occurs [20]. In experimental settings, effective clamping was achieved within a torque range of approximately 1.5 to 2.25 times the threshold torque, with peak clamping at 1.75 times the threshold torque. These findings underline the importance of controlled torque application to achieve reliable fixation while avoiding mechanical failure and stripping of the screw–bone interface [20,21]. In clinical practice, such biomechanical considerations are particularly relevant in foot and ankle surgery, where compression screw constructs are widely employed for fracture fixation, corrective osteotomies, and arthrodesis [22,23,24,25,26]. Reported union and complication rates vary substantially depending on the anatomical site, fixation strategy, and definition of union, as well as patient-related risk factors, surgical complexity, and follow-up duration [22,27]. The MAX Variable Pitch Compression (VPC) Screw System (Zimmer Biomet, Warsaw, IN, USA) is a fully threaded, cannulated, self-tapping, variable-pitch headless screw designed to provide interfragmentary compression for small-bone fixation. However, published clinical data specifically evaluating the MAX VPC Screw System in foot and ankle surgery remain limited. To provide comprehensive real-world clinical data across multiple foot and ankle indications, this post-market retrospective cohort study evaluated union rates, complications, and patient-reported outcomes following fixation of fractures, osteotomies, and arthrodeses using the MAX VPC Screw System.

## 2. Materials and Methods

### 2.1. Study Design and Ethical Approval

This retrospective, single-center cohort study was conducted in accordance with the Declaration of Helsinki [28]. The study protocol was approved by the Northwestern and Central Switzerland Ethics Committee (EKNZ; BASEC ID: 2020-02315) on 8 July 2021. The approval also covered the prospective inclusion and analysis of anonymized patient data collected between 2021 and 2023. All participants provided written informed consent, and data were processed in compliance with the General Data Protection Regulation.

### 2.2. Patient Selection and Eligibility Criteria

All consecutive adult patients who underwent fixation of foot or ankle fractures, corrective osteotomies, or arthrodeses using at least one MAX VPC screw between March 2018 and October 2023 were included. No additional preselection based on anatomical location or procedural complexity was performed within these indication groups, reflecting routine clinical use of the implant. Formal inclusion criteria were age ≥ 18 years and written informed consent. Exclusion criteria included local infection, severe osteoporosis or peripheral vascular disease of the lower extremity, cognitive impairment preventing informed consent or adherence to postoperative care, and any known hypersensitivity to the implant material. Demographic data, comorbidities, surgical details, and postoperative complications were extracted from electronic medical records and entered into a standardized database for analysis.

### 2.3. Surgical Technique and Postoperative Management

All procedures were performed by experienced orthopedic foot and ankle surgeons using standard aseptic technique. The MAX VPC screw (Zimmer Biomet, Warsaw, IN, USA) was inserted under intraoperative imaging guidance according to the manufacturer’s surgical protocol. Screw diameter and length were selected based on bone size and indication. In cases requiring enhanced stability, screws were combined with supplementary fixation to maintain alignment and rotational control. Postoperative management included partial weight-bearing for six weeks. Depending on the indication and soft-tissue status, temporary immobilization was applied using a postoperative shoe, cast, or walker boot. Radiographic follow-up was routinely performed at 6–8 weeks postoperatively and thereafter as clinically indicated, guiding progression to full weight-bearing and range-of-motion exercises based on clinical and radiographic signs of consolidation.

### 2.4. Outcome Measures

Data were collected using predefined case report forms at four time points: (1) enrollment (baseline), (2) intraoperative and immediate postoperative assessment, (3) early follow-up at 6–8 weeks, and (4) long-term follow-up at ≥12 months (1 year or later). At each time point, protocol deviations were documented if applicable. Patient-reported outcomes were collected separately via structured telephone interviews at ≥12 months postoperatively, which marked the completion of the protocol-defined study follow-up.

The primary outcome of this study was bone union or joint fusion, depending on the surgical indication (fracture/osteotomy or arthrodesis). Bone union and fusion were assessed clinically and radiographically at the 6–8-week follow-up and at ≥12 months postoperatively. Radiographic assessments were performed by two experienced orthopedic surgeons who were not blinded to the clinical course. Standard orthogonal radiographs were used for assessment at each time point, with discrepant ratings resolved by consensus. Computed tomography was obtained only when clinically indicated and was not routinely used for union assessment. Union or fusion was defined as evidence of bridging trabeculation across at least three cortices, combined with the absence of pain during functional loading.

Secondary outcomes focused on safety, assessed by recording and analyzing the incidence, type, and frequency of complications and adverse events (AEs), as well as their relationship to the implant, instrumentation, and procedure. All events were classified according to the definitions outlined in EN ISO 14155:2020. For clarity, serious adverse events (SAEs) were defined as serious medical events not directly attributable to the study device, whereas serious adverse device effects (SADEs) referred specifically to implant- or instrumentation-related serious complications, including mechanical failure or loss of fixation. Adverse device effects (ADEs) comprised non-serious device-related events. This classification allowed separate reporting of serious medical events and serious device-related complications. Adverse events were reviewed for severity and causality and documented in a standardized electronic database by the first author.

Performance and clinical benefits were evaluated with the validated Foot and Ankle Ability Measure (FAAM) [29]. FAAM data were collected through structured telephone interviews conducted at least 12 months postoperatively. Interviews were conducted using a standardized script to minimize interviewer influence. The questionnaire consists of two subscales: a 21-item Activities of Daily Living (ADL) scale and an 8-item Sports scale, scored from 0 to 84 and 0 to 32, respectively. Each score was converted to a percentage of the maximum possible value, with higher percentages indicating better functional status.

Range of motion (ROM) was predefined as an exploratory secondary outcome and recorded during routine follow-up visits. In acute fracture cases, preoperative assessment was not feasible due to the nature of the injury.

### 2.5. Statistical Analysis

All eligible procedures treated during the observation period were included in the analysis. A descriptive sample size consideration was informed by previously reported bone union rates of approximately 87% for comparable devices. Based on this assumption, at least 96 evaluable procedures per subgroup (fracture/osteotomy and arthrodesis) were considered necessary. To account for an anticipated loss-to-follow-up rate of approximately 10% over the 12-month follow-up period, the target sample size was increased accordingly, resulting in a planned enrollment of at least 107 procedures per subgroup to ensure an adequate number of evaluable cases for descriptive assessment of clinical outcomes. The sample size consideration was descriptive and not intended to support formal statistical inference. Continuous variables are presented as means with standard deviations (SDs), and categorical variables as absolute and relative frequencies. Bone union, fusion, and complication rates were summarized descriptively. No formal hypothesis testing or inferential statistical comparisons were performed, as the investigation was designed as an exploratory, retrospective post-market clinical follow-up study. All outcomes were analyzed descriptively according to the predefined evaluation criteria in the study protocol. Statistical analyses were performed using R statistical software (Version 4.3.2; R Foundation for Statistical Computing, Vienna, Austria) [30]. Because 51 patients underwent more than one procedure, analyses were conducted at the procedure level, and results should be interpreted descriptively. Missing data were not imputed; analyses were based on available cases at each time point.

### 2.6. Use of Artificial Intelligence

Artificial intelligence-assisted tools (ChatGPT, OpenAI, San Francisco, CA, USA; GPT-4) were used exclusively for language editing and stylistic refinement of the manuscript. No AI tools were used for data analysis, data interpretation, study design, or decision-making processes.

## 3. Results

A total of 214 procedures (165 patients) were included, comprising 107 arthrodeses (50.0%), 80 osteotomies (37.4%), and 27 fracture fixations (12.6%). Radiographic assessment was available in 209/214 procedures (97.7%) at 6–8 weeks and in 82/214 (38.3%) at ≥18 months; FAAM data were obtained for 199/214 procedures (93.0%). Fifty-one patients underwent more than one procedure using the MAX VPC Screw System. The mean patient age was 59.2 ± 12.9 years, and the mean body mass index (BMI) was 25.3 ± 4.3 kg/m^2^. Females accounted for 64.9% of the patients. Concomitant diseases were recorded per patient and are detailed in Table 1. The mean surgical duration was 81 ± 36 min, and the mean length of hospital stay was 2.5 ± 1.6 days. Further details on demographic and surgical characteristics are summarized in Table 1. In fracture/osteotomy procedures, the mean follow-up duration was 17 ± 7 months, with a mean interval of 31 ± 16 months between surgery and FAAM score collection. In arthrodesis procedures, the mean follow-up was 19 ± 8 months, and the mean interval to FAAM assessment was 40 ± 16 months.

### 3.1. Primary Outcome—Joint Fusion and Bone Union

Fracture/osteotomy and arthrodesis union were assessed in 209 procedures (97.7%) at 6–8 weeks and in 82 procedures (38.3%) at ≥18 months postoperatively. The overall union rate was 86.1% at 6–8 weeks, increasing to 98.8% at the final radiographic follow-up. At 6–8 weeks, the union rate was higher in arthrodesis cases (91.5%) compared with fracture and osteotomy cases (80.6%). At ≥18 months, nearly all evaluated procedures (98.8%) achieved radiographic and clinical union. Detailed results by indication and time point are provided in Table 2.

### 3.2. Secondary Outcomes—Complication Rate and PROMs

A total of 28 adverse events (13.1%) were documented, involving 24 patients. Overall, 11 adverse events (5.1%) required re-operation for device removal, whereas the remaining events were managed conservatively. Fourteen adverse events occurred in the fracture/osteotomy group and 14 in the arthrodesis group. Of these, 19 adverse events (67.9%) were classified as device-related, while 9 (32.1%) were unrelated to the implant or instrumentation. The distribution of adverse event categories by indication is provided in Table 3. Among the 11 procedures requiring device removal, the most common indication was nonunion (n = 4, 1.9%), followed by secondary loss of reduction (n = 2, 0.9%). Importantly, no recurrent mechanical failures of the implant were observed throughout the study period. The indications for screw removal are detailed in Table 4.

At a mean follow-up of 2.9 years, FAAM data were available for 199 procedures. In the arthrodesis group (n = 100), the mean FAAM scores were 92.3 ± 14.5 for ADL and 78.8 ± 28.5 for sports. In fracture/osteotomy procedures (n = 99), mean FAAM scores were 94.3 ± 11.0 for ADL and 85.8 ± 20.5 for sports. FAAM outcomes are summarized in Table 5. ROM data were available only for a limited number of procedures and were inconsistently documented across visits; therefore, they are not reported quantitatively.

## 4. Discussion

To the best of our knowledge, this is the first study to evaluate the safety, performance, and clinical benefits of the MAX VPC Screw System in foot and ankle fracture fixation, osteotomies, and arthrodesis. The principal findings were high bone union rates (86.1% at 6–8 weeks and 98.8% at ≥18 months), favorable patient-reported outcomes, and a low overall complication rate with a moderate number of device-related events.

### 4.1. Bone Union and Performance

Primary bone healing requires absolute stability and interfragmentary compression to minimize micromotion and facilitate direct osteonal bridging [1,2]. Variable-pitch headless compression screws achieve this by allowing the proximal fragment to advance more slowly than the distal fragment during insertion, thereby generating compression across the fracture or fusion site [10,12,13,14,15,16,17,18,19]. A recent meta-analysis indicated that fully threaded, tapered headless compression screws were among the designs producing the highest interfragmentary compression forces in a standardized scaphoid fracture model [11]. To our knowledge, this is the first study to evaluate the MAX VPC Screw System in foot and ankle procedures, in which a high early union rate (91.5% at 6–8 weeks), particularly in arthrodesis cases, was found. Although direct comparison is limited by the heterogeneous range of foot and ankle procedures treated in our cohort, the observed outcomes are broadly consistent with the fusion performance reported for screw-based constructs in more uniform models, such as first metatarsophalangeal joint arthrodesis. In this setting, a systematic review by Kang et al. reported a pooled fusion rate of 94.9% for screw fixation [31]. Collectively, these data indicate that the union rates achieved in this cohort fall within the expected range for compression screw techniques. However, reported fusion rates vary considerably depending on study design, anatomical site, and particularly the method used to assess union. In a recent systematic review of ankle, hindfoot, and midfoot arthrodeses, CT-verified fusion rates across heterogeneous clinical cohorts averaged 78.7%, underscoring the substantial impact of imaging modality and definition of union on reported outcomes [27]. The slightly lower early union rate observed in fracture/osteotomy cases (80.6%) may reflect differences in fracture morphology, soft-tissue injury, biological healing potential, and postoperative loading patterns. In addition, the 6–8-week follow-up represents a relatively early radiographic assessment, and union was defined conservatively as bridging trabeculation across at least three cortices. Consequently, incomplete radiographic consolidation at this time point is more likely a reflection of early healing progression rather than delayed union, as nearly all evaluated fracture/osteotomy cases achieved complete radiographic and clinical union at final follow-up. Because fixation strategies in deformity correction vary widely, our findings should be interpreted in the context of the recent literature and alternative stabilization concepts. Restuccia et al. reported percutaneous hallux valgus correction without internal osteosynthesis and no nonunions at mid-term follow-up, demonstrating reliable healing in selected forefoot deformities [32]. Similarly, Colo’ et al. described lateral open-wedge calcaneal osteotomy for adult acquired flatfoot deformity stabilized with temporary K-wire fixation, also reporting low failure rates without a relevant nonunion signal [33]. While alternative fixation concepts have shown favorable healing in selected indications, our findings indicate that rigid compression screw fixation with the MAX VPC Screw System can achieve similarly high union rates across a heterogeneous spectrum of foot and ankle procedures.

### 4.2. Safety and Complications

The overall adverse-event rate in this cohort was 13.1%, and hardware removal was required in 5.1% of procedures, most commonly in the context of nonunion. Direct comparison with published data is limited by the heterogeneous range of foot and ankle procedures included in our cohort. Nevertheless, the cannulated, headless configuration of the MAX VPC screw likely contributes to minimal soft-tissue irritation and a low need for elective implant removal. This is supported by a recent proportional meta-analysis demonstrating hardware-removal rates commonly in the range of 22–28% for headed screws compared with approximately 5–7% for headless constructs following ankle arthrodesis [34]. Importantly, no recurrent mechanical failures were observed in this cohort, suggesting a high degree of structural reliability of the implant under clinical loading conditions.

### 4.3. Quality of Life and PROMs

In the arthrodesis cohort, mean FAAM scores were 92.3 for ADL and 78.8 for Sports, while fracture/osteotomy cases achieved mean scores of 94.3 and 85.8, respectively. These results indicate a good restoration of functional capacity across all groups. Higher Sports subscale scores in fracture/osteotomy cases may be related to preserved joint mobility; however, ROM data were insufficient for meaningful analysis. The mean follow-up of more than 2.5 years supports the durability of these functional improvements beyond the initial healing phase. Although direct comparison is limited by the heterogeneous range of procedures in our cohort, FAAM ADL values were close to published normative reference levels [35], whereas Sports scores were lower in the arthrodesis subgroup, as may be expected after joint fusion.

### 4.4. Strengths and Limitations

Strengths of this study include the large number of patients, clearly defined inclusion criteria, and standardized data collection with systematically documented pre-, intra-, and postoperative parameters. Comprehensive clinical and radiological records were available for most cases, and the extended follow-up period enabled consistent evaluation of outcomes over time. Several limitations must also be acknowledged. The retrospective, single-center design limits causal inference and generalizability, and the absence of a control group precludes direct comparison with alternative fixation techniques. Radiographic assessments were not performed by blinded independent reviewers, which may have introduced observer bias. Because 51 patients underwent more than one procedure, observations are not fully independent, which represents a methodological limitation. The substantially lower long-term (≥18-month) radiographic follow-up rate (38.3% vs. 97.7%) may introduce attrition bias. Furthermore, FAAM was administered via telephone interview, which may introduce interviewer bias and has not been formally validated in this specific mode of administration. Although ROM was predefined as an exploratory outcome, documentation was incomplete and inconsistent across visits, precluding meaningful analysis. While the follow-up duration was sufficient to assess bone union, it was not long enough to capture late complications, implant fatigue, or long-term functional outcomes. These limitations should be considered when interpreting the results, and future prospective, multicenter studies with larger patient cohorts and extended follow-up are warranted to corroborate and expand upon these findings.

## 5. Conclusions

This study demonstrates that the MAX VPC Screw System achieves high union rates with favorable functional recovery in foot and ankle surgery. The moderate incidence of device-related complications supports its favorable safety profile in small-bone fixation of the foot and ankle. However, the retrospective single-center design and limited follow-up duration constrain the generalizability of these findings. Future prospective, multicenter investigations with extended follow-up are warranted to confirm these results and to compare the MAX VPC Screw System directly with alternative fixation techniques.

## Figures and Tables

**Table 1:** Patient Demographics and Surgical Details.

Patient Demographic and Surgical Details	Study Population n = 214
**Age**, mean age in years (±SD)	59.2 (±12.90)
**BMI**, mean in kg/m^2^ (±SD)	25.3 (±4.27)
**Sex**, n (%)	
Female	139 (64.9%)
Male	75 (35.1%)
**Concomitant Disease**, n (%)	
None	117 (70.9%)
Diabetes	10 (6.1%)
Osteoporosis	11 (6.7%)
Vascular disease	21 (12.7%)
Multiple joint disease	6 (3.6%)
Cardiac/Respiratory disease	51 (30.9%)
Nicotine abuse	25 (15.2%)
Autoimmune disorder	5 (3.0%)
Other	30 (18.2%)
**Surgical Indication**, n (%)	
Foot/ankle fracture	27 (12.6%)
Foot/ankle osteotomy	80 (37.4%)
Foot/ankle arthrodesis	107 (50%)
**Surgical Side**, n (%)	
Left	108 (50.5%)
Right	106 (49.5%)
**Surgical Duration**, mean [min] (±SD)	81.14 (±36.23)
**Length of Hospital Stay**, mean [days] (±SD)	2.49 (±1.60)
**Number of Screws Used per Surgery**	
Foot/Ankle Fracture/Osteotomy, n (%)	
1 screw	81 (75.7%)
2 screws	26 (24.3%)
Foot/Ankle Arthrodesis, n (%)	
1 screw	67 (62.6%)
2 screws	35 (32.7%)
3 screws	1 (0.9%)
4 screws	4 (3.7%)

Concomitant diseases are reported at the patient level (n = 165); percentages may exceed 100% due to multiple comorbidities per patient. All other data are reported at the procedure level (n = 214). *n* number; *SD* standard deviation; *min* minutes.

**Table 2:** Assessment of Joint Fusion and Bone Union by Indication After 6–8 Weeks and ≥18 Months.

	Union Rate After 6–8 Weeks n = 209 (97.7%)	Union Rate After ≥18 Months n = 82 (38.3%)
**Foot/Ankle Arthrodesis**	106 (50.7%)	44 (53.7%)
Union	97 (91.5%)	43 (97.7%)
Nonunion	9 (8.5%)	1 (2.3%)
**Foot/Ankle Fracture/Osteotomy**	103 (49.3%)	38 (46.3%)
Union	83 (80.6%)	38 (100%)
Nonunion	20 (19.4%)	0 (0.0%)

Values are reported as absolute counts (%) unless reported otherwise; *n* number.

**Table 3:** Distribution of adverse event categories by indication group (event-level analysis).

	Foot/Ankle Arthrodesis *n* = 14 Events (of 28)	Foot/Ankle Fracture/Osteotomy *n* = 14 Events (of 28)
AE, *n* (%)	2 (7.1%)	1 (3.6%)
SAE, *n* (%)	3 (10.7%)	3 (10.7%)
ADE, *n* (%)	4 (14.3%)	4 (14.3%)
SADE, *n* (%)	5 (17.9%)	6 (21.4%)

*AE* Adverse Event; *SAE* Serious Adverse Event; *ADE* Adverse Device Effect; *SADE* Serious Adverse Device Effect Percentages are calculated using the total number of adverse events (n = 28) as the denominator.

**Table 4:** Reasons for Study Device Removal.

Study Device Removal	*n*, (%)
Device removed as standard of care	2 (0.9)
Device removed upon patient request	1 (0.5)
Device removed due to complication before union/joint fusion	6 (2.8)
Nonunion	4 (1.9)
Intraoperative screw breakage	1 (0.5)
Secondary dislocation	1 (0.5)
Device removed due to complication after bone union/joint fusion	1 (0.5)
Device removed due to revision before bone union/joint fusion	1 (0.5)
**Total devices removed**	**11 (5.1)**

*n* number.

**Table 5:** Quality of Life Assessment Using FAAM After a Mean Follow-Up of 2.9 Years.

	Foot/Ankle Arthrodesis *n* = 100	Foot/Ankle Fracture/Osteotomy *n* = 99
**FAAM**, mean (±SD)		
**ADL Scale**	92.3 ± 14.5	94.3 ± 11.0
**Sports Scale**	78.8 ± 28.5	85.8 ± 20.5

*n* number; *SD* standard deviation; *FAAM* Foot and Ankle Ability Measure; *ADL* Activities of Daily Living.

## Data Availability

The data are available from the corresponding author upon reasonable request but cannot be made publicly available due to privacy and ethical restrictions.

## Abbreviations

The following abbreviations are used in this manuscript:

ADL	Activities of Daily Living
ADE	Adverse Device Effect
AE	Adverse Event
BMI	Body Mass Index
EN ISO	European Norm/International Organization for Standardization
FAAM	Foot and Ankle Ability Measure
MAX VPC	MAX Variable Pitch Compression
PROM	Patient-Reported Outcome Measure
ROM	Range of Motion
SAE	Serious Adverse Event
SADE	Serious Adverse Device Effect
SD	Standard Deviation
VPC	Variable Pitch Compression
